# Supplementary material for: Genotypic and Phenotypic Detection of Polyhydroxyalkanoate Production in Bacterial Isolates from Food
Source: Int J Mol Sci. 2023 Jan 8;24(2):1250. doi: 10.3390/ijms24021250 (PMC9864133; doi:10.3390/ijms24021250)
Supplement: Supplementary file 1 [file ijms-24-01250-s001.zip › ijms-2101885-supplementary.pdf]

International Journal of
Molecular Sciences

MDPI

Supplementary Materials

Article

Genotypic and Phenotypic Detection of Polyhydroxyalkanoate Production in Bacterial Isolates from Food

Daniela Máčalová [1], Magda Janalíková [1], Jana Sedlaříková [2], Iveta Rektoříková [1], Marek Koutný [1] and Pavel Pleva [1,*]

[1] Department of Environmental Protection Engineering, Faculty of Technology, Tomas Bata University in Zlin, 275 Vavreckova, 76001 Zlin, Czech Republic
[2] Department of Fat, Surfactant and Cosmetics Technology, Faculty of Technology, Tomas Bata University in Zlin, 275 Vavreckova, 76001 Zlin, Czech Republic
* Correspondence: ppleva@utb.cz

In this Supplementary Materials, the following table is presented:

Table S1. Identification of strains isolated from different food of plant and animal origin.

	Family	Identification	MALDI score	BLAST similarity (%)	Source
Food of plant origin	Bacillaceae	*Bacillus siamensis*	-	100.00%	garlic
		Bacillus sp.	-	100.00%	romaine lettuce
		Bacillus sp.	-	100.00%	tomato
		Bacillus subtilis	-	99.85%	apple
		Bacillus velezensis	-	100.00%	pepper
		Bacillus velezensis	-	100.00%	onion
		Lysinibacillus fusiformis	-	100.00%	garlic
		Lysinibacillus macroides	-	99.92%	kohlrabi
		Oceanobacillus caeni	-	99.91%	red romaine lettuce
		Oceanobacillus kimchii	2.342	-	red onion
		Peribacillus sp.	-	100.00%	red onion
		Priestia megaterium	-	99.92%	radish
		Priestia megaterium	-	99.92%	romaine lettuce
		Priestia megaterium	-	99.77%	white radish
	Enterobacteriaceae	*Escherichia coli*	-	99.87%	mung bean sprouts
		Escherichia coli	-	99.63%	mung bean sprouts
		Escherichia coli	-	99.91%	spring onion
		Escherichia coli	-	100.00%	zucchini
		Klebsiella oxytoca	2.071	-	white radish
		Lelliottia amnigena	2.284	-	white cabbage
		Pantoea agglomerans	2.256	-	carrot
		Pantoea agglomerans	2.121	-	cucumber

		Pantoea conspicua	-	100.00%	eggplant
		Rahnella aquatilis	2.060	-	leek
	Moraxellaceae	*Acinetobacter calcoaceticus*	-	99.92%	celery stalk
		Acinetobacter calcoaceticus	2.187	-	lettuce
		Acinetobacter calcoaceticus	-	100.00%	lettuce
		Acinetobacter calcoaceticus	2.193	-	white cabbage
		Acinetobacter calcoaceticus	-	99.90%	lettuce
		Acinetobacter calcoaceticus	-	100.00%	lettuce
	Pseudomonadaceae	*Pseudomonas extremorientalis*	2.023	-	white radish
		Pseudomonas marginalis	2.001	-	garlic
		Pseudomonas oryzihabitans	2.176	-	romaine lettuce
		Pseudomonas putida	-	99.91%	chilli pepper
		Pseudomonas sp.	-	99.92%	cucumber
		Pseudomonas sp.	-	99.53%	dill
		Pseudomonas sp.	-	99.92%	kohlrabi
		Pseudomonas sp.	-	99.24%	peach
		Pseudomonas sp.	-	99.91%	leek
	Staphylococcaceae	*Mammaliicoccus sciuri*	-	99.77%	cucumber
		Staphylococcus succinus	-	99.92%	white cabbage
	Xanthomonadaceae	*Stenotrophomonas maltophilia*	-	100.00%	white radish
		Stenotrophomonas rhizophila	-	99.92%	beetroot
Food of animal origin	Enterobacteriaceae	*Escherichia coli*	2.112	-	chicken skin
		Escherichia coli	-	99.12%	duck
		Escherichia coli	-	99.54%	duck
		Escherichia coli	2.035	-	chicken skin
		Escherichia coli	2.244	-	chicken skin
		Escherichia coli	2.177	-	chicken skin
		Escherichia coli	-	99.36%	pheasant
		Escherichia coli	-	100.00%	pheasant
		Escherichia coli	-	99.88%	pheasant
		Escherichia coli	-	99.23%	pheasant
		Escherichia coli	-	100.00%	pheasant
		Escherichia coli	-	99.56%	pheasant
		Escherichia coli	2.036	-	pheasant
		Escherichia coli	2.108	-	pheasant
		Escherichia coli	2.087	-	pheasant
		Escherichia coli	2.229	-	pheasant
	Xanthomonadaceae	*Stenotrophomonas maltophilia*	2.280	-	dairy product
